# Association between sociodemographics and change in alcohol or tobacco use behaviors during the COVID-19 pandemic

**DOI:** 10.1371/journal.pone.0304111

**Published:** 2024-05-31

**Authors:** Selam Habtemariam, Chloe M. Hery, Xiaochen Zhang, Mengda Yu, Darren Mays, Toyin Adeyanju, Brittany Bernardo, Electra D. Paskett, for the Impact of COVID-19 on Behaviors across the Cancer Control Continuum in Ohio group

**Affiliations:** 1 Comprehensive Cancer Center, The Ohio State University, Columbus, Ohio, United States of America; 2 Division of Epidemiology, College of Public Health, The Ohio State University, Columbus, Ohio, United States of America; 3 Fred Hutch Cancer Center, Seattle, Washington, United States of America; 4 Division of Biostatistics, The Ohio State University, Columbus, Ohio, United States of America; 5 The Ohio State University, Columbus, Ohio, United States of America; 6 Denison University, Granville, Ohio, United States of America; 7 Division of Cancer Prevention and Control, Department of Internal Medicine, College of Medicine, The Ohio State University, Columbus, Ohio, United States of America; CDC Foundation, UNITED STATES

## Abstract

**Objective:**

To examine the association between various sociodemographic factors with alcohol and tobacco use behaviors during the COVID-19 pandemic.

**Methods:**

Participants from Ohio and Indiana were asked to participate in the ‘Impact of COVID-19 on the Cancer Continuum Consortium’ study (N = 32,989) from June–November 2020. Those who completed the survey and responded to key study questions were included (n = 5,374). Participants were asked about the frequency and type of alcohol and tobacco product used. Multivariable logistic regression was conducted to determine factors associated with the impact of COVID-19 on change in alcohol and/or tobacco use.

**Results:**

Mean age was 57 years old, 68% were female, 90% non-Hispanic white, 75% married, and 31% lived in rural counties. Out of 5,374 participants, 53% used alcohol-only (n = 2,833), 5% used tobacco-only (n = 255), 7% used both alcohol and tobacco (n = 395), and 35% used neither alcohol nor tobacco (n = 1,891). Urban county of residence (vs. rural) was associated with an increase in alcohol-use (p = 0.0001), change in alcohol products (p = 0.023), and an increase in tobacco use (p = 0.05). Among alcohol-only users, those who were younger (OR = 0.97), female (OR = 1.58), married (OR = 1.69), of high socioeconomic status (OR = 1.99), residing in urban counties (OR = 1.65), and had elevated financial (OR = 1.06) and employment concerns (OR = 1.28) were significantly more likely to report increased alcohol-use. Similarly, among tobacco users, those who were younger (OR = 0.97), female (OR = 2.79), married (OR = 2.16) or divorced (OR = 2.83), and had higher levels of neighborhood disadvantage (OR = 2.19) were significantly more likely to report increased tobacco-use.

**Conclusions:**

Findings suggest targeted intervention and prevention strategies for young, female participants with elevated financial and employment concerns during the COVID-19 pandemic are necessary to mitigate risks associated with higher odds of alcohol and tobacco use. Our findings on alcohol and tobacco use may be a result of the unique social and economic influence of the pandemic on women.

## Introduction

The Coronavirus disease 2019 (COVID-19) pandemic continues to affect populations across the world [1–3]. COVID-19 is a viral respiratory infection with severe symptoms experienced in 15–20% of the population [4]. Though vaccines have been widely accessible in the United States (U.S.), COVID-19 related health concerns remain. Public health strategies have been implemented as a preventive response to contain the disease, including measures for social distancing, quarantining, and stay-at-home-orders [1, 3]. These policies may introduce acute stressors (such as work displacement) and chronic stressors (such as financial instability) [3]. Such stressors thereby have increased general rates of distress [3, 4], feelings of fear and uncertainty [2, 4, 5], anxiety and depression [4–6], feelings of isolation [6], and unhealthy coping behaviors [3–6]. Many of these coping behaviors include addictive behaviors such as increased alcohol and tobacco use that escalate complications due to COVID-19 [7, 8].

Tobacco use rates, including both cigarettes and e-cigarettes, have varied in response to the pandemic [1, 9, 10]. One study found that many tobacco users increased their use during the pandemic, particularly due to stress [1, 11]. Other studies have found that tobacco use has not changed for the majority of smokers internationally due to the pandemic [9, 11]. Conversely, alcohol use has changed over the course of the pandemic with relevant implications in health [2–5]. Psychological distress fueled by COVID-19 is associated with drinking behavior [3], and alcohol consumption was greater among women compared to men [3]. Previous studies identify underlying mechanism of alcohol consumption including using it as an avoidance coping strategy for perceived threats such as stress [3]. Elevated stress levels during the pandemic may result in using alcohol consumption as a maladaptive coping strategy, thereby altering stress response pathways in the hypothalamic–pituitary–adrenal (HPA) axis [12]. This dysregulation can lead to increased dependency on and excessive consumption of alcohol. Moreover, many external factors, such as alcohol sales, may modify alcohol consumption levels.

Despite the shutting down of many bars and stores selling alcohol during the pandemic, there was a 54% increase in sales of alcohol in the U.S. at the peak of the pandemic between March and April of 2020 [2–4]. The greatest increase in alcohol use was in the 35–54 year old age group, with 44% of individuals reporting this change was due to stress [3]. According to another study, 26.4% of participants reported increased alcohol use during the pandemic lockdown. Increased alcohol consumption during the pandemic was often found to be associated with older age, working from home, having children, having higher education, and individuals who consume alcohol in higher quantities [5].

Another crucial consideration is the influence of residential setting on alcohol and tobacco use. Studies prior to the pandemic have found mixed results regarding differences in tobacco and alcohol use behaviors based on U.S. metropolitan county status [13–16]. A previous study found that excessive alcohol use within the past year was not associated with neighborhood socioeconomic status [14]. Tobacco behaviors prior to the pandemic have shown differences in tobacco product type in urban and rural areas [15], but no significant differences in tobacco use practices [13]. However, many of these findings likely have changed with the current social and psychological environment posed by the COVID-19 pandemic.

Socio-economic factors are also associated with substance use, particularly alcohol consumption [5, 17, 18]. Occupation type has been found to be the strongest predictor of frequency of alcohol consumption, while employment status was the weakest [17]. Among all occupational categories, individuals in professional or managerial occupations were found to have greater occasions of drinking, while lower educational attainment was the strongest predictor of binge drinking frequency [17]. Additionally, international studies have found that the amount of alcohol consumed in high or average income individuals was dependent on financial distress [18]. The study found that the influence of alcohol consumption on financial distress was greatest in high income individuals compared to average and low-income individuals. One possible explanation for this was that financial insecurity may have been perceived as a greater threat in higher income individuals compared to their counterparts [18].

Though previous studies have evaluated substance use prior to and during the pandemic, evaluation of demographic factors (e.g., rural or urban residence), are important to include, as geographic setting can affect the risk of alcohol and tobacco use. Therefore, this study aims to examine the relationship between the negative impact of the COVID-19 pandemic in the U.S. on alcohol and tobacco use and the following factors: age, gender, race, socioeconomic status, marital status, financial stress, employment burden, and geographic setting (urban vs. rural residency). These findings are crucial to understand because substance use during the COVID-19 pandemic reveals large implications in both physical and mental health [2–4, 8]. Additionally, excessive alcohol and tobacco use weaken the immune system, thereby increasing susceptibility to and symptoms from the COVID-19 virus [7, 8].

## Methods

### Overview

This study was part of a National Cancer Institute (NCI) funded initiative conducted in conjunction with 16 other NCI-designated Cancer Centers, the IC-4 (Impact of COVID-19 on the Cancer Continuum Consortium) [19]. The initiative was funded to work collectively to develop core survey items and implement population surveys in the respective catchment areas. The overall goal of the IC-4 was to assess how differences in demographics (rural/urban, age, gender, race, educational attainment) impact engagement in cancer preventive behaviors (e.g., tobacco cessation, screening, diet) and cancer management/survivorship behaviors (e.g., adherence to treatment, adherence to surveillance, access to health services) in the context of COVID-19 environmental constraints (e.g., social distancing, employment, mental health). Each site had its own theoretical framework and survey methods. This study was grounded in the Health Belief Model (HBM) [20, 21]. According to the HBM, individuals’ change in health behaviors depends on a series of health beliefs. Our site used the IC-4 core set of common data elements [19], augmented with questions of interest to the local team, with remote data collection methods to include many unique and diverse populations. The survey elements (See S1 Appendix) were finalized in conjunction with other members of the IC-4 [22]. This study was approved by the Ohio State University (OSU) Institutional Review Board in June 2020. All participants provided written, or verbal, informed consent.

### Sample selection

Participants who agreed to be re-contacted from previous studies were asked to participate in this study with a wide variety of populations, including healthy residents and cancer patients. Eligible participants were adults aged 18 years or older who consented to take part in the study. To ensure the inclusion of the most vulnerable, underserved and minority populations, we sought to recruit cancer patients, cancer survivors, and cancer patients and survivors’ caregivers, in addition to healthy adult volunteers, mainly from Ohio, with some from Indiana. This was achieved by employing two recruitment strategies. First, we identified and contacted individuals who previously participated in studies conducted at OSU and consented to be contacted for future research projects. In addition, we invited cancer patients and survivors to nominate their primary caregivers to participate in the study. The list of previous research projects conducted at OSU included the Rural Interventions for Screening Effectiveness (RISE) study (R01 CA196243), the Community Initiative Towards Improving Equity and Health Status (CITIES) cohort (Supplement to P30CA016058), the Buckeye Teen Health Study (BTHS) study (P50CA180908), the Ohio State University Center of Excellence in Regulatory Tobacco Science (CERTS) cohort (P50CA180908), and members of the Total Cancer Care (TCC) cohort (P30CA016058). Second, to further enhance the representative of our study sample and ensure the inclusion of minority and underserved communities, we utilized our community partners and Listservs to send tailored email invitations.

### Interview/Data collection

We utilized several data collection methods, including web, phone, and mailed surveys. Respondents with valid emails received an initial survey invitation email along with three reminders seven days apart. All participants were initially screened using an eligibility form before conducting the survey. Participants were able to save the web survey and resume it at a later time. Those who partially completed the web survey received an email reminder one week after they last accessed the survey. Surveys were estimated to take about 30 minutes. A trained interviewer contacted participants without an email address and those with invalid emails on file by phone. Participants who were initially reached by phone were offered the option to complete the survey over the phone or online. We mailed a cover letter and a paper survey with a self-addressed, stamped return envelope to participants who requested a mailed survey. For Non-English-speaking participants, a bilingual staff member administered the survey in the appropriate language. Participants were offered a $10 gift card upon completion of the survey. All data were collected and managed using the Research Electronic Data Capture (REDCap) secure web-based application hosted at OSU.

### Measures

Self-reported variables of age, sex, race, marital status, geographical setting, socioeconomic status (SES), financial stress, employment burden, and area deprivation index (ADI) were included as predictors in outcome analysis. Race was grouped as: “white,” “Black or African American,” “Asian,” and “Other or multiple.” Levels of marital status were divided into three groups: “Single, never married,” “Married or living as married,” and “Divorced, separated, widowed, or other.” Participants’ counties of residence were classified into urban (metropolitan) and rural (nonmetropolitan) on the basis of the 2013 Rural-Urban Continuum Codes (RUCC) [23]. Counties with RUCCs of 1–3 were coded as urban/metro, while those with RUCCs of 4–9 were coded as rural/nonmetro.

SES was estimated with the Hollingshead Four Factor Index of Socioeconomic Status [24] using variables of income, insurance, education, and occupation. Income was derived from responses that were grouped into four categories: <$35,000; $35,000-$49,999; $50,000-$74,999; and ≥$75,000. Income score ranged from 0 to 3, with the lowest income range scored at 0, and the highest at 3. Insurance was grouped into four categories which each corresponded with a score: no insurance, public-only, private-only, and both public and private. Insurance score ranged from 0 to 2, with no insurance scored at 0, public-only at 1, and private-only or public and private at 2. Education was grouped into four categories: high school or less, some college/Associate degree, Bachelor’s degree (e.g. BS/BA), and Master’s degree or higher (e.g. MS, PhD). Education score ranged from 0 to 3, with high school or less scored at 0, and Master’s degree or higher scored at 3. Occupational status groups included employed full/part time, retired, and other (e.g., disabled, unemployed). It was scored from 0 to 2, with “other” scored at 0, retired scored at 1, and full or part time employment at 2. Each of these values were represented in an estimate SES between 0 (lowest SES) and 10 (highest SES).

Financial stress score was calculated using the following variables: financial loss, housing, providing for family, and concern for food and supplies. Questions that were used to assess these variables included the following: (1) “During the last 30 days, how often have you worried about personal financial loss (e.g., lost wages, job loss, investment/retirement loss, travel-related cancellations)?” (2) “During the last 30 days, how often have you worried about making rent or mortgage payments?” (3) “How much have you worried about providing for yourself or your family?” (4) “During the last 30 days, how often have you worried about providing for yourself or your family?” (5) “During the last 30 days, how often have you worried about not having enough basic supplies such as household cleaning supplies or toilet paper?” For each of the five questions, patient chose one of the following responses that were summed for the financial stress score: (0) none of the time (1) some of the time or (2) most of the time, or (3) all of the time. The summary score for financial stress ranged from 0–15 (with 0 being lowest concern and 15 being highest concern).

Additionally, an employment burden score was calculated using a summary of variables including job loss, job payment status, and concern of losing a job due to COVID-19. The following questions assessed these variables respectively: (1) “Since March 1st, did you lose a job because of COVID-19?” where participants responded either “Yes (1)” or No (0)”; (2) “Are you currently being paid for a full or part-time job, including being paid by a job while you stay home? Do not include unemployment compensation” where participants answered either “Yes (1)” or “No (0)”; (3) “How concerned are you about losing your job due to COVID-19?” where participants answered either “Not at all/slightly concerned (0)” or “Somewhat/moderately/extremely concerned (1).” A summary score of employment burden ranged from 0–3 (with 0 being lowest concern and 3 being highest concern).

Participant addresses were geocoded to a point location based on street address, then joined to block groups from the 2010 US Census Tiger file [25]. The 2015, block-group-level, Area Deprivation Index (ADI) was linked to participants’ geocoded 2010 block group [26]. The 2015 ADI uses the 2011–2015 American Community Survey variables to generate a national ranking of ADI values [27]. The ranking ranges from percentiles of 1% to 100%, with higher values representing more disadvantaged ADI. We categorized ADI percentile rankings into quartiles for descriptions of the study population and used a two group cutoff in our multivariate analyses to describe the highest 20% of the sample living in disadvantaged areas (≤80% vs >80%).

### Outcomes

Self-reported alcohol and tobacco use was categorized based on responses to questions pertaining to frequency of use and type of products used.

Questions pertaining to tobacco use included “Have you changed the frequency of tobacco or marijuana use compared to before the COVID-19 pandemic?” with responses including “Yes, I have used tobacco or marijuana products more,” “Yes, I have used tobacco or marijuana products less,” and “No, I have been using the same amount of tobacco or marijuana.” Participants were asked “Which product(s) did you begin using in the past 30 days?” in which they selected all products that applied including cigarettes, electronic cigarettes, cigars, and other tobacco containing products. Those who reported products relating to marijuana (e.g., e-cigarettes containing marijuana) were not included in the analysis (n = 320).

Questions pertaining to alcohol use included “Have you changed the amount of alcohol you drink compared to before the COVID-19 pandemic?” with responses including “Yes, I have drunk more alcohol,” “Yes, I have drunk less alcohol,” and “No, I have drunk the same amount.” Type of alcohol consumption was assessed through the question “Have you changed the type of alcohol (beer, wine, liquor, etc.) you drink compared to before the COVID-19 pandemic?” in which participants answered either “Yes” or “No.”

### Data analysis

The descriptive statistics used to summarize characteristics included means with standard deviation for continuous variables and frequencies with percentages for categorical variables. Participant characteristics were compared using Chi-squared tests for categorical variables and t-tests and ANOVA for continuous variables. Demographic summaries were also compared by geographical residential setting (urban vs. rural counties). Outcomes associated with tobacco and alcohol use behaviors were analyzed in relation to the predictors of interest (age, gender, race, marital status, SES, urban vs rural county, financial stress score, employment burden score, and ADI). Multivariable logistic regression was used to assess the impact of COVID-19 on 1) change in type of alcohol products and tobacco products, and 2) increase in alcohol consumption and tobacco consumption.

All statistical analyses were conducted using SAS v9.4 (SAS Institute, Cary, NC, USA), with a significance level of 0.05.

## Results

### Study population

**Fig 1:**
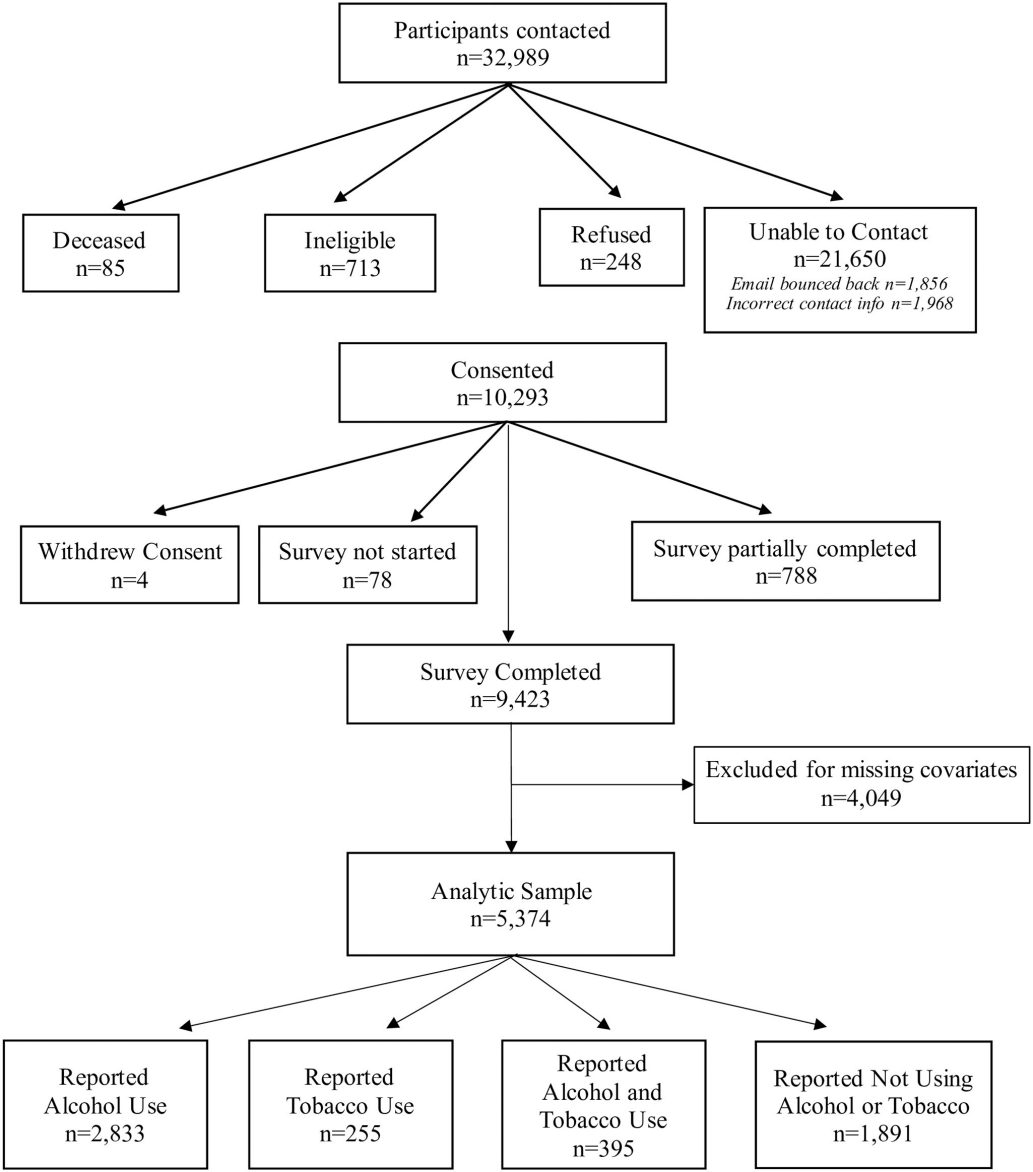
COVID-19 survey recruitment diagram.

A total of 32,989 participants were approached during recruitment efforts. Participants were excluded if researchers were unable to contact them (n = 21,650), or they were ineligible (n = 713), refused (n = 248), or were deceased (n = 85). Of the 32,989 originally contacted, 10,293 were consented (33.8% response rate). Participants who withdrew consent (n = 4), did not start the survey (n = 78), or only partially completed the survey (n = 788) were excluded. Therefore, a total of 9,423 participants completed the survey (Fig 1). The current analysis only included participants who had complete responses to all the variables analyzed and were grouped into the following four categories: alcohol-only users (53%, n = 2,833), tobacco-only users (5%, n = 255), alcohol and tobacco users (7%, n = 395), and non-users of alcohol and tobacco (35%, n = 1,891), for a total sample of 5,374.

Of all the participants, most were between the ages of 41–60 years old (45%, n = 2,437) and 68% (n = 3,646) were female (Table 1). Most participants were white (89.7%, n = 4,821), married or living as married (74.9%, n = 4,023), and from the middle SES level (41.5%, n = 2,231). There were significant differences between the demographic characteristics of the four substance use groups. Notably, participants ≤40 years old (n = 102, 25.8%) were more likely to be both alcohol and tobacco user, as were male participants (n = 186, 47.1%). However, female participants were more likely to be alcohol-users only (n = 1,961, 69.2%) or were non-users of both (n = 1,319, 69.8%).

**Table 1:** Demographic summary of survey participants by alcohol-only use (n = 2,833), tobacco-only use (n = 255), alcohol and tobacco use (n = 395) and non-users of alcohol and tobacco use (n = 1,891).

Variable	Total (n = 5,374)	Alcohol-only Use (n = 2,833, 53%)	Tobacco-only Use (n = 255, 5%)	Alcohol & Tobacco Use (n = 395, 7%)	Non-Users of Alcohol & Tobacco (n = 1,891, 35%)	P-value
**Age**, years, mean ± SD	56.56 ± 12.94	56.21 ± 12.61	53.6 ± 12.37	51.27 ± 13.31	58.6± 13.01	<0.001
**Age Group**, N (%)						<0.001
≤40 years old	707 (13.2%)	359 (12.7%)	43 (16.9%)	102 (25.8%)	203 (10.7%)	
41–60 years old	2437 (45.4%)	1360 (48.0%)	135 (52.9%)	182 (46.1%)	760 (40.2%)	
>60 years old	2230 (41.5%)	1114 (39.3%)	77 (30.2%)	111 (28.1%)	928 (49.1%)	
**Sex**, N (%)						<0.001
Male	1728 (32.2%)	872 (30.8%)	98 (38.4%)	186 (47.1%)	572 (30.3%)	
Female	3646 (67.9%)	1961 (69.2%)	157 (61.6%)	209 (52.9%)	1319 (69.8%)	
**Race**, N (%)						<0.001
White	4821 (89.7%)	2603 (91.9%)	208 (81.6%)	349 (88.4%)	1661 (87.8%)	
Black/African American	320 (5.95%)	134 (4.73%)	34 (13.3%)	24 (6.08%)	128 (6.77%)	
Asian	89 (1.66%)	28 (0.99%)	2 (0.78%)	5 (1.27%)	54 (2.86%)	
Other/Multiple	144 (2.68%)	68 (2.40%)	11 (4.31%)	17 (4.30%)	48 (2.54%)	
**Marital Status**, N (%)						<0.001
Single, never married	457 (8.5%)	198 (7.0%)	51 (20.0%)	50 (12.7%)	158 (8.4%)	
Married/living as married	4023 (74.9%)	2212 (78.08%)	140 (54.9%)	284 (71.9%)	1387 (73.4%)	
Divorced/Widowed Separated/Other	894 (16.6%)	423 (14.9%)	64 (25.1%)	61 (15.4%)	346 (18.3%)	
**SES group**^1^, N (%)						<0.001
0–5	1140 (21.2%)	326 (11.5%)	157 (61.6%)	114 (28.9%)	543 (28.7%)	
6–8	2231 (41.5%)	1176 (41.5%)	69 (27.1%)	174 (44.1%)	812 (42.9%)	
9–10	2003 (37.3%)	1331 (47.0%)	29 (11.4%)	107 (27.1%)	536 (28.3%)	
**Financial Stress Score**^2^, N (%)						<0.001
Median ± SD (min, max)	2.54 ± 3.11 (0, 15)	2.14 ± 2.65 (0, 15)	4.82 ± 4.29 (0, 15)	3.27 ± 3.72 (0, 15)	2.67 ± 3.24 (0, 15)	
**Employment Burden Score**^3^, N (%)						<0.001
Median (SD) (min, max)	0.64 ± 0.69 (0, 3)	0.71 ± 0.68 (0, 3)	0.47 ± 0.7 (0, 3)	0.68 ± 0.72 (0, 3)	0.56 ± 0.69 (0, 3)	
**County of Residence**^4^, N (%)						<0.001
Rural	1665 (31.0%)	803 (28.3%)	100 (39.2%)	115 (29.1%)	647 (31.0%)	
Urban	3709 (69.0%)	2030 (71.7%)	155 (60.8%)	280 (70.9%)	1244 (69.0%)	
**ADI Level**^5^, N (%)						< .0001
≤80	4680 (87.1%)	2579 (91.0%)	173 (67.8%)	334 (84.6%)	1594 (84.3%)	
>80	694 (12.9%)	254 (9.0%)	82 (32.2%)	61 (15.4%)	297 (15.7%)	
**ADI Quartile**^5^, N (%)						< .0001
Quartile 1 (0–34)	1490 (27.7%)	968 (34.2%)	18 (7.1%)	90 (22.8%)	414 (21.9%)	
Quartile 2 (35–52)	1351 (25.1%)	745 (26.3%)	57 (22.4%)	103 (26.1%)	446 (23.6%)	
Quartile 3 (53–71)	1302 (24.2%)	623 (22.0%)	68 (26.7%)	98 (24.8%)	513 (27.1%)	
Quartile 4 (>71)	1231 (22.9%)	497 (17.5%)	112 (43.9%)	104 (26.3%)	518 (27.4%)	

^1^Lowest SES (0–5) to highest SES (9–10); calculated with income, insurance, education, and occupation variables.

^2^Summary score calculated with frequency of concerns for financial loss, housing, family, food, and basic supplies. Ranges from 0–15 (with 0 being lowest concern and 15 being highest concern).

^3^Summary score calculated with job loss, job pay, and concern of losing job. Ranges from 0–3 (with 0 being lowest concern and 3 being highest concern).

^4^Metropolitan County identified by Rural-Urban Continuum Codes (RUCC 2013)

^5^Area Deprivation Index (ADI) ranges from 1–100%, with higher percentiles representing higher levels of disadvantage.

A greater proportion of white participants were alcohol-only users (n = 2,603, 91.9%) and a greater proportion of Black/African American participants were tobacco-only users (n = 34, 13.3%). Those who were single or never married were more likely to be tobacco-only users (n = 51, 20.0%) than alcohol-only, both, or non-users. Additionally, participants from the lowest SES group level (n = 157, 61.6%), those with higher financial stress scores (4.82± 4.29), and those who live in rural counties (n = 100, 39.2%) were all more likely to be tobacco-only users than alcohol-only users, both, or non-users. Participants from the highest ADI quartile (most disadvantaged neighborhoods) were also most likely to be tobacco-only users (n = 112, 43.9%).

### Association of geographic setting and substance use during COVID-19

Over 5% of participants reported a change in the type of alcoholic drink consumed (n = 157, 5.5%) and 23.5% (n = 665) reported an increase in alcohol consumption compared to before the COVID-19 pandemic among alcohol-only users (Table 2). Geographic setting (rural vs. urban county residents) was significantly associated with alcohol use. More urban residents reported a change in the type of alcohol product used compared to rural residents (6.2% vs 4.0%, p = 0.023) and reported increased alcohol consumption compared to rural residents (26.1% vs 16.8%, p<0.0001).

**Table 2:** Change in alcohol or tobacco use behaviors in rural vs. urban residents during the COVID-19 pandemic (among alcohol-only users and tobacco-only users).

**Variable**	**Level**	**Total (n = 2833)**	**Rural residents (n = 803; 28.3%)**	**Urban residents (n = 2030; 71.7%)**	**P-value**
**Alcohol Use (among alcohol-only users)**					
Change in type of alcohol product during COVID-19 pandemic	Yes	157 (5.5%)	32 (4.0%)	125 (6.2%)	0.023
	No	2676 (94.5%)	771 (96.0%)	1905 (93.8%)	
Increased alcohol consumption compared to before the COVID-19 pandemic	Yes	665 (23.5%)	135 (16.8%)	530 (26.1%)	< .0001
	No	2168 (76.5%)	668 (83.2%)	1500 (73.9%)	
**Variable**	**Level**	**Total (n = 255)**	**Rural residents (n = 100; 39.2%)**	**Urban residents (n = 155; 60.8%)**	**P-value**
**Tobacco Use (among tobacco-only users)**					
Change in type of tobacco product during COVID-19 pandemic	Yes	12 (4.7%)	6 (6.0%)	6 (3.9%)	0.185
	No	243 (95.3%)	94 (94.0%)	149 (96.1%)	
Increased tobacco use compared to before the COVID-19 pandemic	Yes	58 (22.8%)	17 (17.0%)	41 (26.5%)	0.05
	No	197 (77.2%)	83 (83.0%)	114 (73.4%)	

Approximately 5% (n = 12) of participants reported a change in the type of tobacco product used and over 22% (n = 58) reported they had increased tobacco use compared to before the COVID-19 pandemic among tobacco-only users (Table 2). There was a significant difference in the increase of tobacco use by urban and rural residents (26.5% vs 17.0%, respectively, p = 0.05), but not for change in product type.

Among participants who reported both alcohol and tobacco use (Table 3), geographic setting was not associated with changes or increases in frequency of alcohol use or tobacco use.

**Table 3:** Change in alcohol or tobacco use behaviors in rural vs. urban residents during the COVID-19 pandemic (among alcohol and tobacco users group).

Variable	Level	Total (n = 395)	Rural residents (n = 115; 29.1%)	Urban residents (n = 280; 70.9%)	P-value
**Alcohol Use (among alcohol and tobacco users)**					
Change in type of alcohol product during COVID-19 pandemic	Yes	39 (9.9%)	14 (12.2%)	25 (8.9%)	0.32
	No	356 (90.1%)	101 (87.8%)	255 (91.1%)	
Increased alcohol consumption compared to before the COVID-19 pandemic	Yes	117 (29.6%)	28 (24.4%)	89 (31.8%)	0.14
	No	278 (70.4%)	87 (75.6%)	191 (68.2%)	
**Tobacco Use (among alcohol and tobacco users)**					
Change in type of tobacco product during COVID-19 pandemic	Yes	22 (5.6%)	7 (6.1%)	15 (5.4%)	0.77
	No	373 (94.4%)	108 (93.9%)	265 (94.6%)	
Increased tobacco or marijuana use compared to before the COVID-19 pandemic	Yes	127 (32.1%)	33 (28.7%)	94 (33.6%)	0.35
	No	268 (67.9%)	82 (71.3%)	186 (66.4%)	

### Association of sociodemographic characteristics and substance use during COVID-19

#### Alcohol use behaviors

Older participants, who reported alcohol-only use or both alcohol and tobacco use, were significantly less likely to change the type of alcoholic drink and less likely to increase their alcohol consumption (Table 4). Female participants who reported alcohol-only use had 1.58 times the odds of increased alcohol consumption during COVID-19 compared to male participants (OR: 1.58, 95% CI: 1.28, 1.96). Black/African American participants had about two times the odds of changing the type of alcohol they drank during the pandemic compared to white participants (OR: 2.07, 95% CI: 1.12, 3.82). Asian participants had lower odds of increasing their alcohol consumption compared to white participants (OR: 0.32, 95% CI: 0.11, 0.94). We also observed those who were married had higher odds of increased alcohol consumption among the alcohol-only users compared to those who were single (OR: 1.69, 95% CI: 1.16, 2.47). Those of the highest SES group also had higher odds of changing their type of alcohol used (OR: 1.99, 95% CI: 1.02, 3.83) and increasing their alcohol consumption compared to those from the lowest SES group (OR: 1.99, 95% CI: 1.34, 2.94). Participants who resided in urban counties and reported alcohol-only use had higher odds of increasing their alcohol consumption compared to those in rural counties (OR: 1.65, 95% CI: 1.32, 2.07). Additionally, participants with higher financial stress scores had higher odds of changing their alcohol type (OR: 1.11, 95% CI: 1.05, 1.17) and increasing alcohol consumption among those that were alcohol-only users (OR: 1.06, 95% CI: 1.02, 1.10). Participants with higher employment burden scores were also more likely to increase their alcohol consumption in the alcohol-only users group (OR: 1.28, 95% CI: 1.10, 1.48). Area Deprivation Index (ADI) was not associated with change of alcohol types or increasing alcohol consumption during COVID-19.

**Table 4:** Association between sociodemographic characteristics and change of alcohol use during COVID-19 pandemic, among alcohol-only users (n = 2,833) and alcohol & tobacco users (n = 395), multivariable adjusted.

	Alcohol-Only Users		Alcohol & Tobacco Users	
Outcome	Change of Alcohol Type	Increased Alcohol Consumption	Change of Alcohol Type	Increased Alcohol Consumption
	Odds Ratio (95% CI)	Odds Ratio (95% CI)	Odds Ratio (95% CI)	Odds Ratio (95% CI)
**Age** (continuous)	**0.98 (0.96, 0.99)**	**0.97 (0.96, 0.98)**	**0.96 (0.93, 0.99)**	**0.96 (0.94, 0.98)**
**Sex**				
Male	ref	ref	ref	ref
Female	0.96 (0.66, 1.39)	**1.58 (1.28, 1.96)**	1.55 (0.73, 3.29)	1.52 (0.93, 2.49)
**Race**				
White	ref	ref	ref	ref
Black/African American	**2.07 (1.12, 3.82)**	0.74 (0.46, 1.19)	0.27 (0.03, 2.25)	0.52 (0.16, 1.72)
Asian	2.12 (0.70, 6.46)	0.32 (0.11, 0.94)	—	0.41 (0.04, 4.18)
Other/Multiple	0.91 (0.34, 2.39)	0.74 (0.41, 1.33)	1.11 (0.23, 5.44)	1.71 (0.56, 5.22)
**Marital Status**				
Single, never married	ref	ref	ref	ref
Married/living as married	0.68 (0.40, 1.16)	**1.69 (1.16, 2.47)**	1.08 (0.38, 3.03)	1.86 (0.85, 4.08)
Divorced/Widowed/Separated/Other	0.74 (0.38, 1.45)	1.28 (0.82, 2.01)	1.18 (0.32, 4.36)	1.84 (0.71, 4.80)
**SES Group** ^1^				
0–5	ref	ref	ref	ref
6–8	1.41 (0.74, 2.66)	1.37 (0.94, 2.00)	0.70 (0.29, 1.66)	1.02 (0.55, 1.90)
9–10	**1.99 (1.02, 3.89)**	**1.99 (1.34, 2.94)**	0.58 (0.20, 1.65)	**2.15 (1.06, 4.39)**
**County of Residence** ^2^				
Rural	ref	ref	ref	ref
Urban	1.20 (0.78, 1.83)	**1.65 (1.32, 2.07)**	0.83 (0.40, 1.75)	1.41 (0.82, 2.41)
**Financial Stress Score** ^3^	**1.11 (1.05, 1.17)**	**1.06 (1.02, 1.10)**	1.03 (0.96, 1.13)	1.06 (0.99, 1.13)
**Employment Burden Score** ^4^	1.08 (0.83, 1.39)	**1.28 (1.10, 1.48)**	1.32 (0.79, 2.19)	0.95 (0.66, 1.37)
**ADI** ^5^				
≤80	ref	ref	ref	ref
>80	1.13 (0.64, 2.00)	0.86 (0.60, 1.23)	0.91 (0.35, 2.36)	0.65 (0.31, 1.35)

^1^ Lowest SES (0–5) to highest SES (9–10); calculated with income, insurance, education, and occupation variables

^2^ Metropolitan County identified by Rural-Urban Continuum Codes (RUCC 2013)

^3^ Summary score calculated with frequency of concerns for financial loss, housing, family, food, and basic supplies. Ranges from 0–15 (with 0 being lowest concern and 15 being highest concern).

^4^ Summary score calculated with job loss, job pay, and concern of losing job. Ranges from 0–3 (with 0 being lowest concern and 3 being highest concern).

^5^ADI ranges from 1–100%, with higher percentiles representing higher levels of disadvantage.

#### Tobacco use behaviors

Older participants who were both alcohol and tobacco users were significantly less likely to increase tobacco use in comparison to younger participants (OR: 0.97, 95% CI: 0.96, 0.99, Table 5). Compared to male participants, female participants who were both alcohol and tobacco users had almost three times the odds of increased tobacco use during the pandemic (OR = 2.79, 95% CI: 1.71, 4.56). Among those who were both alcohol and tobacco users, those who were married (OR: 2.16, 95% CI: 1.01, 4.62) or divorced/widowed/separated (OR: 2.83, 95% CI: 1.15, 7.00) had higher odds of increased tobacco use during the pandemic compared to those who were single. Also, among those who were users of both alcohol and tobacco, urban residents had higher odds of increased tobacco use compared to rural residents (OR: 1.70, 95% CI: 1.00, 2.89). Participants with higher financial stress scores had higher odds of increasing their tobacco use in both groups (tobacco-only users [OR:1.10, 95% CI:1.02, 1.19] and alcohol and tobacco users [OR:1.07, 95% CI: 1.00, 1.14]). Finally, those who were users of both alcohol and tobacco and resided in areas of high disadvantage had over two times the odds of increased tobacco use (OR: 2.19, 1.16, 4.15). Race, SES, and employment burden score were not associated with either a change of tobacco product or increasing tobacco use during the COVID-19 pandemic.

**Table 5:** Association between sociodemographic characteristics and change of tobacco use during COVID-19 pandemic, among tobacco-only users (n = 255), and alcohol & tobacco users (n = 395), multivariable adjusted.

	Tobacco-only Users		Alcohol & Tobacco users	
Outcome	Change of Tobacco Type during Pandemic	Increased Tobacco Consumption during Pandemic	Change of Tobacco Type during Pandemic	Increased Tobacco Consumption during Pandemic
	Odds Ratio (95% CI)	Odds Ratio (95% CI)	Odds Ratio (95% CI)	Odds Ratio (95% CI)
**Age** (continuous)	0.96 (0.91, 1.02)	1.00 (0.97, 1.03)	0.98 (0.95, 1.02)	**0.97 (0.96, 0.99)**
**Sex**				
Male	ref	ref	ref	ref
Female	1.02 (0.26, 4.03)	1.39 (0.68, 2.83)	1.01 (0.39, 2.61)	**2.79 (1.71, 4.56)**
**Race**				
White	ref	ref	ref	ref
Black/African American	5.10 (0.78, 32.98)	0.33 (0.09, 1.13)	—	0.55 (0.20, 1.56)
Asian	—	—	—	1.13 (0.16, 7.89)
Other/Multiple	3.17 (0.29, 34.07)	2.44 (0.67, 8.90)	1.45 (0.17, 12.61)	0.89 (0.28, 2.77)
**Marital Status**				
Single, never married	ref	ref	ref	ref
Married/living as married	8.80 (0.76, 102.00)	0.56 (0.21, 1.45)	0.37 (0.10, 1.39)	**2.16 (1.01, 4.62)**
Divorced/Widowed/Separated/Other	1.43 (0.07, 26.87)	0.96 (0.35, 2.67)	2.00 (0.49, 8.21)	**2.83 (1.15, 7.00)**
**SES Group** ^1^				
0–5	ref	ref	ref	ref
6–8	0.71 (0.13, 3.93)	0.73 (0.29, 1.80)	1.94 (0.56, 6.64)	1.21 (0.67, 2.18)
9–10	0.58 (0.05, 7.02)	1.03 (0.31, 3.43)	1.86 (0.43, 8.01)	1.14 (0.56, 2.34)
**County of Residence** ^2^				
Rural	ref	ref	ref	ref
Urban	0.65 (0.17, 2.47)	1.44 (0.71, 2.95)	0.84 (0.31, 2.27)	**1.70 (1.00, 2.89)**
**Financial Stress Score** ^3^	0.96 (0.81, 1.14)	**1.10 (1.02, 1.19)**	1.03 (0.90, 1.17)	**1.07 (1.00, 1.14)**
**Employment Burden Score** ^4^	0.24 (0.05, 1.24)	1.41 (0.86, 2.32)	1.03 (0.53, 1.99)	1.02 (0.71, 1.45)
**ADI** ^5^				
≤80	Ref	Ref	Ref	Ref
>80	0.24 (0.04, 1.27)	0.55 (0.25, 1.18)	0.51 (0.10, 2.50)	**2.19 (1.16, 4.15)**

¹ Lowest SES (0–5) to highest SES (9–10); calculated with income, insurance, education, and occupation variables

^2^ Metropolitan County identified by Rural-Urban Continuum Codes (RUCC 2013)

^3^ Summary score calculated with frequency of concerns for financial loss, housing, family, food, and basic supplies. Ranges from 0–15 (with 0 being lowest concern and 15 being highest concern).

^4^ Summary score calculated with job loss, job pay, and concern of losing job. Ranges from 0–3 (with 0 being lowest concern and 3 being highest concern).

^5^ADI ranges from 1–100%, with higher percentiles representing higher levels of disadvantage.

## Discussion

This study examined the association of sociodemographics (e.g., age, sex, race, marital status, SES, geographic setting, financial stress, employment burden, ADI) with changes in alcohol and tobacco use during the COVID-19 pandemic. Our study highlighted an increase of alcohol use and tobacco use in more than a fifth of respondents during the COVID-19 pandemic. Social factors that particularly increased the odds of alcohol use included being younger, female, married, and living in an urban area. Economic factors that increased the frequency of alcohol use included higher SES, higher financial stress, and higher employment burden. For tobacco, factors that increased odds of use included being younger, female, married or divorced, having higher financial stress, and living in an area of high disadvantage.

Our study found that urban residence was significantly associated with increases in both alcohol and tobacco use. Compared to rural residents, urban residents reported significantly higher rates of changes in type of alcohol product, increasing alcohol consumption, and increasing tobacco use. Though research on the association between geographic setting and substance use during the pandemic is limited, these findings are somewhat in line with studies conducted prior to the pandemic on tobacco use [15]. Underpinning the risk of these behaviors may be feelings of uncertainty and isolation caused partially by stress and restrictive measures during the pandemic [1–6, 10]. Additionally, changes in sales of alcohol and tobacco during the pandemic in urban versus rural areas may influence access to the substances, but must be investigated further [2–4].

### Alcohol use behavior implications

Contrary to our hypothesis, younger participants were more likely than older participants to report an increase in the amount of alcohol and to change the type of alcohol product used during the pandemic. These findings are similar to previous studies that found that older adults were at a lower risk for reporting increased alcohol during the COVID-19 pandemic [28–30]. Previous studies suggest that younger individuals have been detrimentally impacted by restricted social gatherings and have the highest levels of mental distress during the pandemic [28, 29].

Our study also highlighted that female participants had higher odds of increasing alcohol consumption compared to their male counterparts. Qualitative interviews from previous studies similarly revealed the unique, gendered effects of the pandemic on stress and alcohol use [28, 29, 31]. They found women experienced greater mental health instability, feelings of loneliness, caregiving responsibilities [29], and unpaid work, in comparison to men during the pandemic [31]. Women saw an increase in perceived value of consuming alcohol as a coping mechanism [29, 31], a decrease in their likelihood of reducing alcohol use in comparison to men [28], and significant increases in moderate or severe alcohol use disorder during the COVID-19 pandemic [30]. These experiences are crucial when considering women’s health.

Additionally, our findings revealed that Black/African American participants had two times the odds of changing the type of alcohol during the pandemic compared to white participants. Another study found significant changes in alcohol use behaviors (increased daily drinking and alcohol use disorder) in Black/African American participants during the pandemic [30]. Though it was unclear why changes in drinking behavior occurred in Black/African American participants, the previous study suggests this may be due to disproportionate rates of economic distress, mortality due to COVID-19, and increased stress as a result of events such as the 2020 killings of Breonna Taylor and George Floyd by U.S. law enforcement.

Other sociodemographic factors that influenced alcohol use in our study were marital status, financial stress, and employment burden. Married participants reported higher odds of increased alcohol consumption. Additionally, greater financial stress and employment burden were associated with change in alcohol product type used and an increase in consumption during the pandemic, as supported by other studies [29, 32, 33]. Our findings are consistent with previous findings on unemployment and self-employment being unique drivers of increased substance use due to increased economic and social instability influencing businesses [29].

Interestingly, individuals with higher SES in our study also had two times the odds of reporting a change in and an increase in alcohol use during the pandemic in comparison to adults of lower SES. This is consistent with a European study that found that those with higher income reported increased alcohol use, particularly if they also reported financial distress [33]. Other studies report mixed findings, with residents of the highest education levels and lowest income reporting the smallest levels of regular alcohol use [34], while another study found that SES wholistically had no significant influence [14]. An explanation for our findings may be due to elevated stress from jobs with high responsibility which likely increased during the pandemic as suggested by a previous study [29]. Another study on alcohol sales during the pandemic interestingly found that respondents decreased their household expenditure on alcohol and tobacco [35]. While expenditures may predict alcohol and tobacco consumption, a limitation identified in the study was that frequency of use and the possibility of cheaper substitutions of products were not explored.

### Tobacco use behavior implications

Our study found that younger participants were more likely to increase tobacco use compared to older participants. These findings are consistent with previous research that found participants older than 55 years of age were at lower risk for increasing tobacco use during the pandemic compared to younger participants [28].

Our study also determined female participants also had almost three times the odds of increasing tobacco use among users of both alcohol and tobacco. In line with our findings, a previous study that used data from the eastern WHO European Region found women had higher odds of reporting elevated tobacco use during the pandemic compared to men [28]. Research also suggests that women had greater caregiver stress that uniquely increased as a result of the pandemic [29, 31]. Similar to alcohol use, our study’s findings on tobacco use in women may be explained by gender convergence as a result of the unique social and economic influence of pandemic on women [3]. Increased tobacco use was also found in individuals of higher financial stress in our study. This is consistent with the findings of another study that also found that areas with higher unemployment rates and lower income are at high risk for tobacco use; interestingly, these factors had the most effect on women in these studies [29].

### Strengths & limitations

Several limitations of our study should be addressed. Generalizability of our findings are limited to mainly to the Ohio and Indiana area. The sample size of recruited participants that met eligibility criteria for this study was also not large, in comparison to some COVID-19 survey studies, or nationally representative enough. Additionally, the strict stay-at-home orders, quarantining, and social distancing measures in place during the time of the distribution of the survey used for this study likely impacted responses collection. However, the survey response rate was acceptable for this study, likely indicating that recruitment methods worked effectively. Answers to questions in the survey were also self-reported and likely are subject to recall bias. Another shortcoming of this study includes the wording of survey questions about the time period to consider. Some questions limited participants to respond on their behaviors within the past 30 days only, including financial stress and substance use. An additional consideration is the timing of our survey. The survey was conducted from June to November 2020, during the first year of the COVID pandemic and therefore participant behavior (e.g., substance use) may not represent their current behaviors. Finally, previous studies have found that psychological distress was significantly associated with the frequency of alcohol use behaviors to cope with the pandemic [2–5, 31], and general poorer mental health outcomes (high anxiety, depression, and feelings of loneliness) that influence both addictive behavior outcomes [2, 6]. However, mental health or ongoing diagnosed mental health conditions of the participants were not analyzed in this study.

Despite these limitations, this study has several notable strengths, including a comprehensive survey covering many questions on alcohol and tobacco use. As a result, this study addresses many gaps in the literature including that of changes in type of alcohol and tobacco substances used. Limited research was available on users of multiple substances (alcohol and tobacco) during the pandemic which was addressed thoroughly in this study. The influence of geographic setting, financial stress, and employment burden on substance use during the COVID-19 pandemic were also uniquely analyzed, addressing other gaps in the literature. Finally, the survey was administered from June 19, 2020 through November 30, 2020, providing data for a crucial window of time during the pandemic. The survey was also administered by email, mail, and phone which allowed us to reach as broad a geographic distribution as possible.

### Conclusions

Our study found that female participants, individuals younger than 40 years of age, and urban residents were most likely to increase both alcohol and tobacco use during the pandemic. Additionally, those of higher SES, those experiencing higher financial stress and higher employment burden, and married participants were more likely to increase their consumption of alcohol during the pandemic. Addressing substance use behaviors during a time of collective societal stress and major lifestyle changes is crucial. Understanding these stressors should be topics of future research. Our findings suggest the need for prevention and intervention strategies on substance use during the COVID-19 pandemic, whether that be through telehealth cessation strategies or other methods. Additionally, greater emphasis on interventions in more vulnerable communities, including women, younger individuals, and communities experiencing increased financial and employment struggles throughout this public health crisis is important to combat the alcohol and tobacco dependence disparities suggested by the results. Further research should be directed towards monitoring the trends of alcohol and tobacco use over time, particularly with a larger sample size. Additional data on alcohol and tobacco sales might also be needed in the future to confirm the validity of similar findings. Finally, following up with participants to explore the motives behind increasing substance use during the pandemic is important to understand causality of increased substance use as seen in other studies [11].

## Supporting information

S1 Appendix Survey elements by core constructs. (DOCX)
